# Vendor and software based variation in left atrial strain measurements: implications for clinical practice

**DOI:** 10.1007/s10554-025-03499-3

**Published:** 2025-09-03

**Authors:** Vlad Danaila, Oliver Archer, Luke Stefani, Aaisha Ferkh, Shaun Khanna, Faraz Pathan, Paula Brown, Liza Thomas

**Affiliations:** 1 Department of Cardiology, Westmead Hospital, Western Sydney Local Health District, Parramatta, NSW Australia; 2 Westmead Clinical School, University of Sydney, Sydney, NSW Australia; 3 Department of Cardiology, Blacktown Hospital, Western Sydney Local Health District, Parramatta, NSW Australia; 4 Department of Cardiology, Nepean Hospital, Nepean Blue Mountains Local Health District, Parramatta, NSW Australia; 5 South West Clinical School, University of New South Wales, Sydney, Australia; 6 Department of Cardiology, PO Box 533, Wentworthville, NSW 2145 Australia

**Keywords:** Echocardiography, Left atrial strain, Intervendor agreement

## Abstract

Left atrial strain (LAS) is a valuable echocardiographic marker of left atrial function with growing clinical utility. However, variability in LAS measurements across software vendors remains a barrier to its routine clinical use. This study aimed to compare LAS measurements obtained using a dedicated left atrial-specific measurement tool (AFI-LA (General Electric (GE)) with those derived from established LV-based strain platforms: GE EchoPAC (mid-myocardial and endocardial tracking) and TomTec-Arena (feature tracking), used for measurement of LAS. LAS was measured in 112 subjects (72 individuals in sinus rhythm and 40 paroxysmal atrial fibrillation (PAF) patients in sinus rhythm at the time of transthoracic echocardiography). Reservoir (ƐR), contractile (ƐCT), and conduit (ƐCD) phasic strain were measured using AFI-LA and compared with GE-mid, GE-endo, and TomTec strain measurements. Agreement was assessed using Bland–Altman analysis and Pearson correlation. Inter- and intra-observer reproducibility was evaluated. AFI-LA measurements showed good correlation with both platforms (*r* ≥ 0.7, *p* < 0.001). AFI-LA consistently underestimated strain values across all phases compared to GE-mid, GE-endo, and TomTec, with the smallest bias observed against GE-mid. Proportional bias was present, particularly at higher strain values. Inter- and intra-observer reproducibility of AFI-LA measurements was high (*r* > 0.85). AFI-LA provides LAS measurements that are most comparable to GE mid-myocardial strain but demonstrates systematic underestimation compared to GE endocardial and TomTec-derived values. These differences underscore the need for vendor-specific calibration before AFI-LA can be used for serial assessments or applied using existing clinical threshold values. Larger validation studies are needed to support standardization and broader clinical adoption.

## Introduction

Left atrial strain (LAS) is a robust measure of left atrial function, and alterations in LAS often precede left atrial (LA) enlargement [1]. LAS has increasing clinical relevance, particularly in evaluation of diastolic function, atrial fibrillation (AF), heart failure and stroke [2–6]. More recently, LAS has demonstrated utility in identifying individuals with elevated left ventricular filling pressures [7]. Standardization of LAS measurements is, therefore, crucial to improving its utilisation in clinical practice for risk stratification and for guiding treatment decisions. Vendors offer various algorithms for measuring myocardial strain, and the ‘strain standardization initiative’ [8] improved intervendor correlation for left ventricular (LV) strain measurements, demonstrating a strong correlation (*r* > 0.8) between vendors, thereby increasing the use of LV strain in clinical practice. However, routine use of LAS in clinical practice has hitherto been limited due to the lack of a dedicated LAS measurement package and the unaddressed issue of intervendor variability for LAS.

The techniques to evaluate two-dimensional (2D) strain rely on semi-automated software packages using speckle-tracking echocardiography or edge tracking using feature-tracking imaging. However, until recently, all LAS measurements were performed using software packages developed to evaluate LV strain. The two most common vendor platforms for LAS, which utilise an LV strain package, include 2D speckle tracking of the mid-myocardium using General Electric (GE) EchoPAC, and the endocardial feature-tracking algorithm using TomTec (TomTec Arena).

A dedicated LA specific automated function imaging (AFI-LA) software tool (GE) for the evaluation of LAS using 2D speckle tracking has recently been developed which is likely to increase the routine clinical uptake of LAS. AFI-LA permits automated 2D speckle-tracking measurements of real-time myocardial deformation. However, almost all the published literature to date, including the determination of normal LAS values, has used the 2 LV strain packages mentioned above. In order to utilise the AFI-LA derived LAS in routine clinical practice, it is essential to evaluate its performance compared with existing LV strain-based LAS measurements. Hence, we sought to compare AFI-LA derived LAS measurements with two existing software platforms where LAS was measured using an LV strain evaluation package: (1) 2D speckle-tracking endocardial and mid-myocardial LAS using GE EchoPAC (General Electric, Milwaukee, WI, USA), and (2) the endocardial feature-tracking based TomTec-Arena software (TomTec Imaging Systems GmbH, Unterschleissheim, Germany).

## Methods

### Study population

The study enrolled adult volunteers (*n* = 72) in sinus rhythm (SR) from Westmead Hospital (Sydney, Australia) from a departmental database comprising healthy controls and individuals with cardiovascular risk factors but no prior cardiac events. We additionally enrolled 40 consecutive patients with paroxysmal atrial fibrillation (PAF) who were in sinus rhythm at the time of transthoracic echocardiogram (TTE) and had left atrial (LA) dilatation (LA indexed volume > 34 ml/m²). These patients were previously recruited to an AF study between 2018 and 2019. Exclusion criteria included a history of ischaemic or structural heart disease, cardiomyopathy, or significant valvular disease.

The study ethics approval was obtained from the Human Research Ethics Committee of Western Sydney Local Health District (HREC/17/WMEAD/435).

## Echocardiographic protocol

All TTEs were performed by experienced cardiac sonographers on two commercially available ultrasound systems, GE Vivid E95 and Vivid E9 (General Electric, Milwaukee, WI, USA), in accordance with American Society of Echocardiography (ASE) guidelines [9]. Echocardiographic images of the parasternal long and short axis and apical four-, two- and long axis were obtained. Focused and optimised apical views of the left atrium were acquired and stored at high frame rates (≥  55 frames per second). Traditional LV and LA volume measurements were recorded as per ASE guidelines [10]: LV end-diastolic volume (LVEDV) and end-systolic volumes (LVESV), LV ejection fraction (LVEF), LA maximum volume at end systole (LAVmax), and LA minimum volume at end diastole (LAVmin). Measurements of LA function included left atrial emptying fraction (LAEF) and left atrial function index (LAFI) [11].

Cine loops of three cardiac cycles were acquired for strain analysis. LAS was measured independently by a trained and experienced investigator, blinded to whether subjects were healthy volunteers or patients with paroxysmal AF. The novel AFI-LA software was used for LAS measurements (GE Echopac (v203, Milwaukee, WI, USA)) and was compared to the LAS measurements obtained using the two LV strain packages of 2D speckle-tracking endocardial, mid-myocardial (conventional) GE Echopac (version 203) and the feature-tracking based TomTec-Arena 2D Cardiac Performance Analysis software (version 2.30.02, TomTec Imaging Systems GmbH, Unterschleissheim, Germany).

**Fig. 1:**
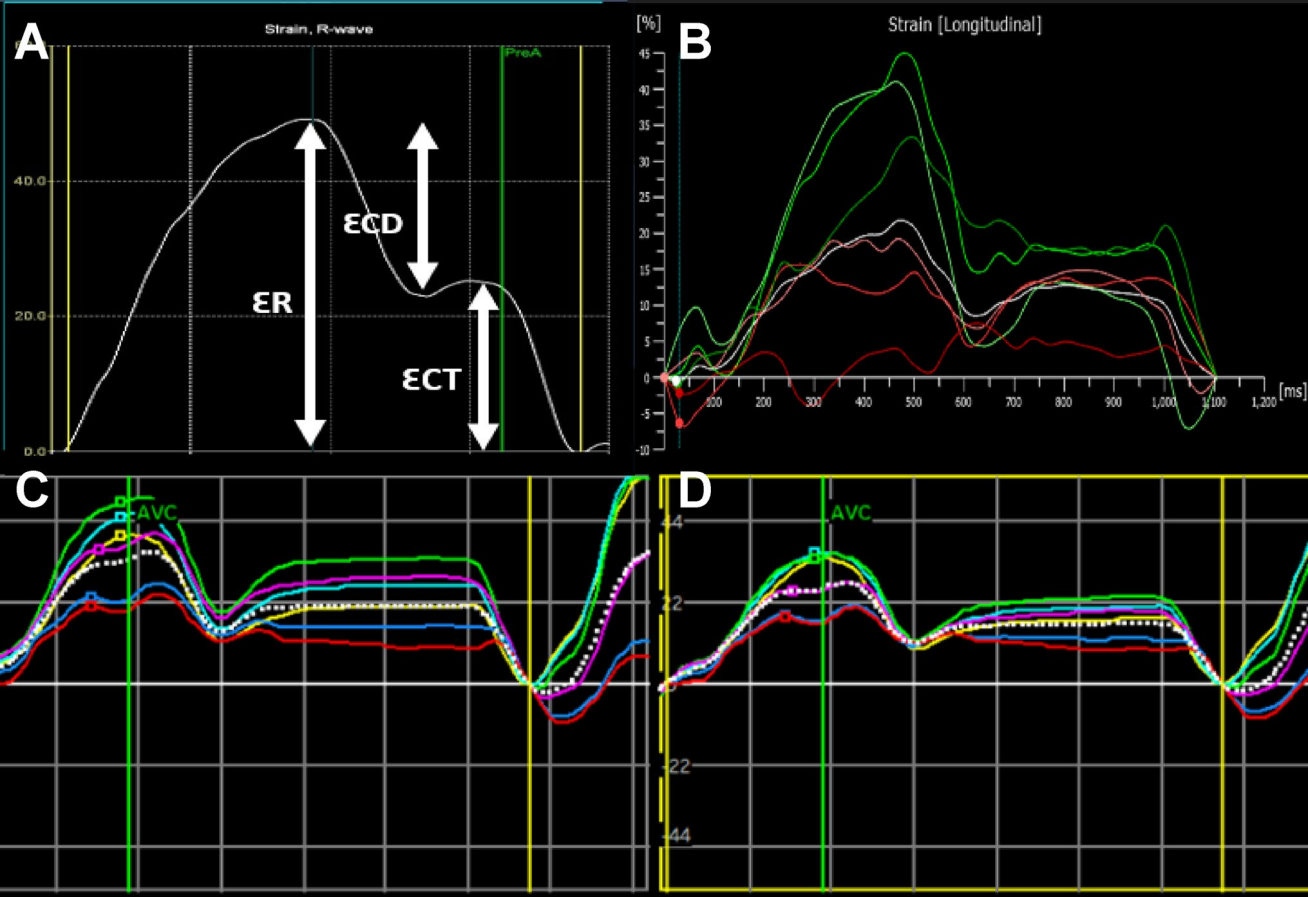
Left atrial strain contour with R-R gating using various vendor and software packages; Picture A: GE Echopac Automated Function Imaging (AFI-LA); Picture B: TomTec endocardial feature tracking package; Picture C: GE Echopac Endocardial 2D speckle tracking; Picture D: GE Echopac mid-myocardial 2D speckle tracking. ƐR: left atrial reservoir strain, ƐCT: left atrial contractile strain, ƐCD: left atrial conduit strain, GE: General Electric

The LA endocardial surface was manually traced using a point-and-click approach. The epicardial surface tracing was automatically generated by the system to obtain a region of interest (ROI), which was adjusted to the thickness of the LA wall. The ROI was traced across the endocardial surface of the LA from the medial to the lateral mitral valve (MV) annulus in the apical 4-chamber view, and from the inferior to anterior MV annulus in the apical 2-chamber view. R-R gating was used for the strain analysis. Strain curves were generated and were subsequently used to determine reservoir strain (ƐR), contractile strain (ƐCT), and conduit (ƐCD) strain (Fig. 1). Three cine loops of the echocardiographic data were analysed separately and averaged.

Inter- and intra-observer reproducibility for LAS using TomTec and GE LV packages and AFI-LA measurements was evaluated in 10 randomly selected subjects. Two repeated measurements of each variable were performed on these subjects by two blinded investigators (LS and VD).

### Statistical analysis

Continuous variables are presented as mean ± standard deviation (SD) and analysed using a two-sample t-test. Categorical values are shown as percentages and frequencies and analysed using chi-squared test. Baseline demographic data and echocardiographic measurements are reported for the SR and PAF groups. Bland–Altman plots were used to illustrate the systematic bias and limits of agreement (LOA) between LV strain-derived TomTec and GE mid- and endocardial measurements for ƐR, ƐCT, and ƐCD, compared with AFI-LA measurements. The mean difference (MD), representing systematic bias, and 95% LOA were calculated for each pairwise comparison. Proportional bias was assessed by regressing the difference between methods against the mean of the two measurements, with a significant slope indicating proportional bias. While the Bland–Altman plots visually display proportional bias using regression lines with 95% confidence intervals, MD and LOA were assessed numerically. Pearson’s correlation coefficient (r) was also calculated to quantify the strength of linear association between AFI-LA and LV strain-derived algorithms which were applied to the LA. The inter- and intra-observer correlations and the coefficients of variance (COV) between repeated measurements are reported along with the associated Bland–Altman analyses. IBM SPSS Statistics version 29 (SPSS, Chicago, IL, USA) was used to analyse the data.

## Results

### Baseline characteristics and TTE data

There were 72 patients in SR and 40 PAF patients in SR at the time of their echocardiogram; the majority were male (*n* = 80, 76%). Healthy volunteers were younger and had a lower BMI compared to those in the PAF group (mean age 47.4 vs. 63.5 years, *p* < 0.001; BMI 26.9 vs. 29.6 kg/m^2^, *p* = 0.001) (Table 1).

**Table 1:** Baseline characteristics of healthy volunteers and patients with paroxysmal atrial fibrillation (PAF)

	Sinus Rhythm (*n* = 72)	PAF (*n* = 40)	*p*-value
Male	55 (76%)	25 (63%)	0.091
Female	17 (24%)	15 (38%)	0.180
Age (years)	47.4 ± 15.3	63.5 ± 7.8	< 0.001
BMI (kg/m²)	26.9 ± 4.8	29.6 ± 4.9	0.005
BSA (m²)	1.9 ± 0.2	2.0 ± 0.2	0.313

Categories are displayed as mean ± standard deviation (SD) for continuous variables and n (%) for categorical variables. BMI: body mass index, BSA: body surface area. P-value represents pairwise comparisons between sinus rhythm and paroxysmal atrial fibrillation groups using a two-sample t-test for continuous variables and a chi-square test for categorical variables

There was no significant difference in LVEF between the two groups (59.6 ± 7.2% in sinus rhythm vs. 56.8 ± 11.0% in PAF, *p* = 0.490). Patients with PAF demonstrated significantly larger LVEDV compared with those in sinus rhythm (106 ± 36.8 mL vs. 91.1 ± 27.3 mL, *p* = 0.008), with a similarly significant difference in indexed LVEDV, albeit within normal limits (54.3 ± 20.1 mL/m² vs. 46.9 ± 12.5 mL/m², *p* = 0.009). Although LVESV and its indexed values were larger in the PAF group, this did not reach statistical significance (*p* = 0.246 and *p* = 0.270, respectively).

Patients in the PAF group had larger LAVmax and LAVmin volumes. Similarly, LA function parameters of LAEF (46.1 ± 14.6% vs. 55.5 ± 10.1%, *p* < 0.001) and LAFI (25.0 ± 12.0 vs. 44.3 ± 13.6, *p* < 0.001) were also reduced in the PAF group (Table 2).

**Table 2:** Baseline echocardiographic data of healthy volunteers and patients with paroxysmal atrial fibrillation (PAF)

	Sinus Rhythm	Paroxysmal AF	*p*-value
LVEDV (mL)	91.1 ± 27.3	106.0 ± 36.8	0.008
LVEDV indexed (mL/m^2^)	46.9 ± 12.5	54.3 ± 20.1	0.009
LVESV (mL)	41.7 ± 43.9	47.0 ± 27.1	0.246
LVESV indexed (mL/m^2^)	21.6 ± 23.5	24.1 ± 15.4	0.270
LVEF Biplane (%)	59.6 ± 7.2	56.8 ± 11.0	0.490
LAVmin (mL)	22.6 ± 7.1	47.4 ± 15.0	< 0.001
LAVmax (mL)	51.0 ± 12.4	88.5 ± 21.6	< 0.001
LAVmax Indexed (mL/m^2^)	26.4 ± 5.9	45.3 ± 10.3	< 0.001
LAEF (%)	55.5 ± 10.1	46.1 ± 14.6	< 0.001
LAFI (mL/m^2^)	44.3 ± 13.6	25.0 ± 12.0	< 0.001

Categories are displayed as mean ± standard deviation (SD). LVEDV: left ventricular end-diastolic volume, LVESV: left ventricular end-systolic volume, EF: ejection fraction, LAEDV: left atrial end-diastolic volume, LAESV: left atrial end-systolic volume, LA Max Indexed: left atrial maximum volume indexed to body surface area, LAEF: left atrial emptying fraction, LAFI: left atrial function index. P-value represents pairwise comparisons between sinus rhythm and paroxysmal atrial fibrillation groups using a two-sample t-test

All phasic left atrial strain components were significantly reduced in the PAF group across all software platforms and tracking methods (*p* < 0.001 for all comparisons). The largest absolute differences were observed in reservoir strain, but consistent reductions were evident across contractile and conduit phases, confirming impaired phasic LA function in PAF patients regardless of vendor (Table 3).

**Table 3:** Comparison of phasic left atrial strain (ƐR, ƐCT, ƐCD) measured using GE EchoPAC (mid-myocardial and endocardial), GE AFI-LA, and TomTec software in healthy volunteers and patients with paroxysmal atrial fibrillation (PAF)

	Sinus Rhythm	Paroxysmal AF	*p*-value
GE Endo ƐR (%)	42.6 ± 8.2	26.9 ± 7.3	< 0.001
GE Mid ƐR (%)	35.2 ± 6.5	22.8 ± 5.9	< 0.001
TomTec ƐR (%)	41.1 ± 6.4	28.4 ± 7.5	< 0.001
GE AFI ƐR (%)	32.9 ± 7.2	19.9 ± 5.6	< 0.001
GE Endo ƐCT(%)	17.9 ± 4.3	11.5 ± 5.0	< 0.001
GE Mid ƐCT (%)	16.4 ± 3.9	10.6 ± 4.5	< 0.001
TomTec ƐCT (%)	17.1 ± 4.4	11.7 ± 5.0	< 0.001
GE AFI ƐCT (%)	14.0 ± 3.7	9.0 ± 4.3	< 0.001
GE Endo ƐCD (%)	24.7 ± 7.4	15.4 ± 4.1	< 0.001
GE Mid ƐCD (%)	18.8 ± 6.1	12.1 ± 3.3	< 0.001
TomTec ƐCD (%)	24.0 ± 6.1	16.6 ± 4.6	< 0.001
GE AFI ƐCD (%)	18.9 ± 6.9	10.8 ± 3.3	< 0.001

Categories are displayed as mean ± standard deviation (SD). ƐR: left atrial reservoir strain, ƐCT: left atrial contractile strain, ƐCD: left atrial conduit strain, GE: General Electric, AFI: Automated Function Imaging. P-value represents pairwise comparisons between sinus rhythm and paroxysmal atrial fibrillation groups using a two-sample t-test

### Inter-vendor agreement vs. AFI-LA derived LA strain measurements

The bias (mean difference), correlation (r) and Bland-Altman LOA between AFI-LA and TomTec, GE-mid and GE-endo measurements for each LAS variable are presented in Table 4. There was good correlation between AFI-LA vs. GE-mid, GE-endo, and TomTec feature tracking strain across all phasic LA strain measurements [*r* ≥ 0.7 for all comparisons (*p* < 0.001)].

**Table 4:** Correlation, mean difference and limits of agreement: GE AFI vs. TomTec, GE mid and GE Endo left atrial strain values

Strain Type	Comparison of AFI vs.	*r*	MD	*p*-value	Lower LOA	Upper LOA
ƐR	TomTec	0.749	−8.3	< 0.001	−21.0	4.4
	GE mid	0.769	−2.5	< 0.001	−14.4	9.4
	GE endo	0.752	−8.8	< 0.001	−23.0	5.5
ƐCT	TomTec	0.737	−2.9	< 0.001	−10.1	4.2
	GE mid	0.755	−2.1	< 0.001	−8.6	4.5
	GE endo	0.749	−3.4	< 0.001	−10.6	3.8
ƐCD	TomTec	0.748	−5.4	< 0.001	−14.9	4.2
	GE mid	0.794	−0.4	0.147	−8.9	8.0
	GE endo	0.776	−5.4	< 0.001	−15.2	4.4

Categories are displayed as correlation coefficient (r), mean difference (MD), and limits of agreement (LOA). ƐR: left atrial reservoir strain, ƐCT: left atrial contractile strain, ƐCD: left atrial conduit strain, GE: General Electric, endo: endocardial. P-value represents pairwise comparisons between AFI and other measurement methods using a two-sample t-test

**Fig. 2:**
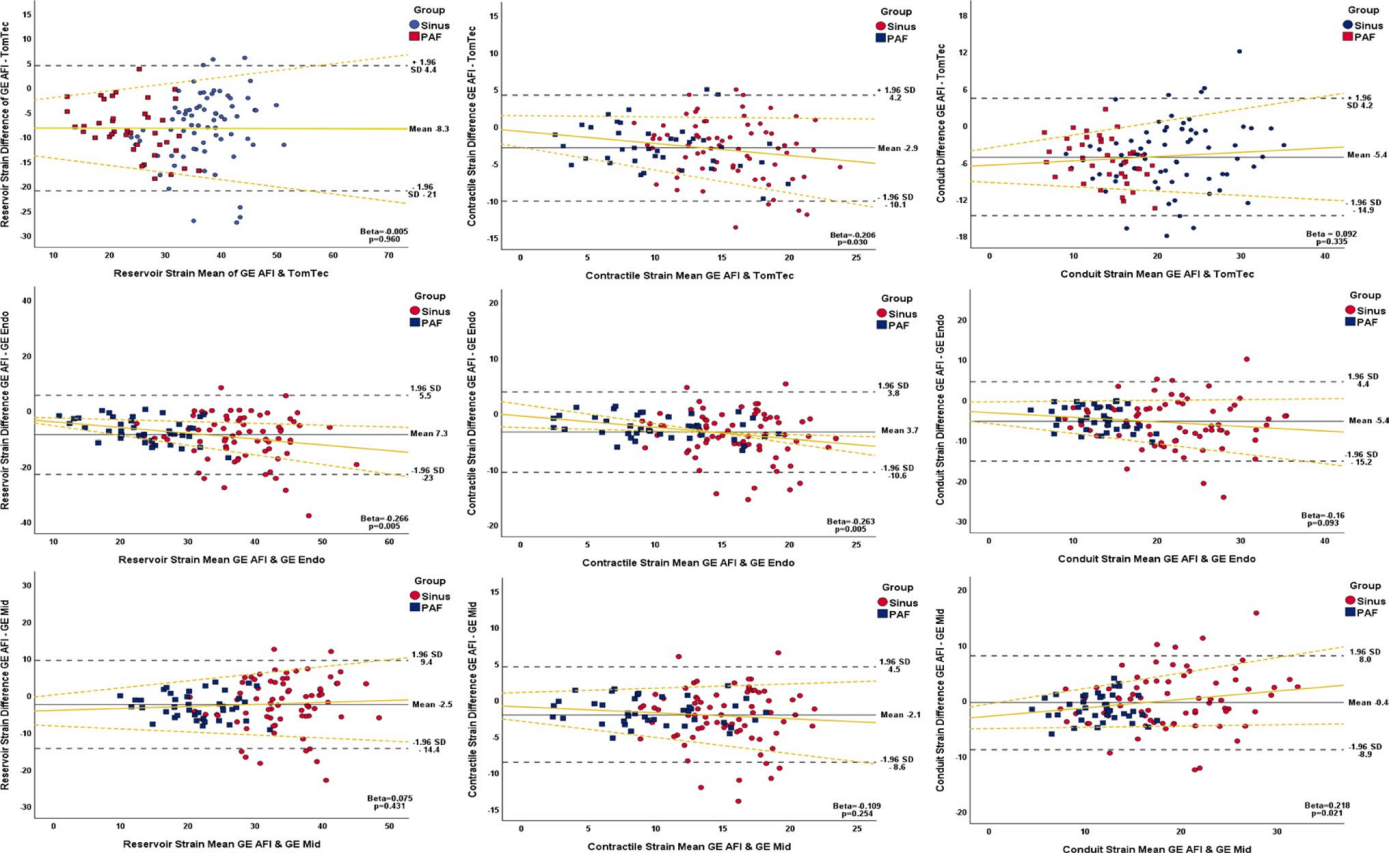
Bland-Altman plots demonstrating the relationship between GE AFI LAS and 2D speckle-tracking GE endocardial, GE mid-myocardial and feature-tracking TomTec measurements of reservoir (ƐR), contractile (ƐCT) and conduit (ƐCD) strain. The solid yellow line represents proportional bias, with corresponding 95% confidence interval (yellow dotted lines) and respective beta and p-values annotated. Sinus: sinus rhythm group; PAF: paroxysmal atrial fibrillation group

Bland–Altman analysis (Fig. 2) demonstrated a systematic bias, with AFI-LA consistently underestimating all phasic strain values compared with LV packages of TomTec, GE-mid, and GE-endo derived LA strain measurements. The smallest bias and narrowest limits of agreement were observed between AFI-LA and GE-mid strain values, indicating better agreement. In contrast, the largest bias and greatest variability were seen in comparisons with TomTec, particularly for ƐR.

Proportional bias was observed in several comparisons, with significant regression slopes in the Bland–Altman plots suggesting that underestimation by AFI-LA increased with higher LA strain values. This effect was most evident for AFI-LA ƐCT and ƐCD strain when compared with TomTec and GE-endo measurements. These findings were consistent across both rhythm groups, although greater variability was noted in the PAF group, especially for ƐR.

There was good intra- and inter-observer correlation for AFI-LA derived LAS measurements with correlation exceeding 0.85 (*p* < 0.001 for all values, Table 5).

**Table 5:** Intra-observer and inter-observer reproducibility for left atrial strain measured with GE Echopac automated function imaging (AFI)

Strain Type	Correlation (*r*)	*p*-value	Coefficient of variation (%)	Mean Difference (MD)	SD	*p*-value	Lower LOA	Upper LOA
*Intra-observer (observation 1 vs. 2)*								
ƐR GE AFI	0.971	< 0.001	0.1	−0.4	3.0	0.282	−6.4	5.6
ƐCT GE AFI	0.860	< 0.001	6.2	0.4	2.2	0.228	−3.9	4.7
ƐCD GE AFI	0.975	< 0.001	0.9	−0.4	0.6	0.251	−5.5	4.7
*Inter-observer LS vs. VD*								
ƐR GE AFI	0.99	< 0.001	0.9	1.1	2.0	0.013	−2.8	4.9
ƐCT GE AFI	0.935	< 0.001	3.0	0.7	1.9	0.052	−3.0	4.4
ƐCD GE AFI	0.988	< 0.001	3.1	0.4	1.7	0.155	−3.0	3.8

Categories are displayed as correlation coefficient (r), mean difference (MD), coefficient of variation, standard deviation (SD), and limits of agreement (LOA). ƐR: left atrial reservoir strain, ƐCT: left atrial contractile strain, ƐCD: left atrial conduit strain, GE: General Electric, endo: endocardial. P-value represents pairwise comparisons using a two-sample t-test

## Discussion

Our analysis demonstrates that the LA specific AFI-LA package has good correlation with LA phasic measurements derived using two LV strain packages (used for LA strain assessment). A small yet systematic underestimation was noted for all LA phasic strain, with the smallest underestimation observed between GE-mid LAS and AFI-LA phasic strain measurements. Interobserver reproducibility was excellent regardless of the software package used.

There is increasing interest in LAS as a clinical tool for the evaluation of diastolic function, LV filling pressure, and as a prognostic marker for cardiovascular disease [2–4, 7, 12]. Most reported studies have utilised an LV strain package for 2D speckl-tracking of the mid-myocardium using General Electric (GE) EchoPAC or the endocardial feature-tracking algorithm using TomTec (TomTec Arena). These algorithms have been utilised to derive normal values of phasic LAS as well as cutoff values for LA strain in various disease states. More recently, a LA specific AFI-LA package has been developed to increase use in routine clinical practice. The AFI-LA software algorithm is specific for the LA and is easy to use. It has also demonstrated good reproducibility, with strong inter- and intra-observer correlation. However, no systematic comparisons have been performed between the LV strain packages and the AFI-LA package.

While the newer AFI-LA is designed specifically for LA strain evaluation, significant differences exist in phasic strain values when compared with GE (mid and endo) and TomTec LV strain measurements applied to the LA. These differences highlight the need for software-specific reference ranges. Moreover, they indicates that LAS measurements across vendors and software platforms cannot be used interchangeably, particularly in the longitudinal follow-up of patients.

Previous studies [13, 14], including work from our group, have compared the widely used 2D mid-myocardial LAS from GE EchoPAC with endocardial LAS from GE EchoPAC and TomTec Arena. Both vendors participated in the European Association of Cardiovascular Imaging—American Society of Echocardiography (ASE) initiative to standardize LV strain imaging. Data collected using both systems have contributed to a meta-analysis defining normal LAS values. These studies [13, 14] demonstrated small but significant differences between mid-myocardial and endocardial LAS derived from 2D speckle-tracking (GE) and feature-tracking strain (TomTec), with speckle tracking generally yielding lower values. Our results build on this evidence, showing that AFI-LA derived LAS measurements are most comparable to GE mid-myocardial values, while larger systematic bias exists when compared with GE endocardial and TomTec LAS. These findings reinforce the need for calibration strategies to improve the standardization of LAS measurements across various platforms.

The variation in LAS across different vendor systems has important clinical implications. Variability in LAS measurements may affect risk stratification and management in various clinical conditions including atrial fibrillation, heart failure, diastolic dysfunction, and stroke risk assessment [5, 15–17]. LAS has also demonstrated an emerging role in valvular heart disease, cardiotoxicity monitoring, and congenital heart disease, with reproducible measurements necessary for longitudinal evaluation and prognostication in these instances [12, 18, 19]. Serial measurements performed using different software packages could lead to inconsistent measurements and misclassification of atrial dysfunction, and impact clinical decision-making. These discrepancies also raise concerns regarding the applicability of current LAS cutoffs used in clinical practice. For instance, British Society of Echocardiography guidelines [20] recommend LAS thresholds for elevated LV filling pressures of < 18% for sinus rhythm and < 20% for atrial fibrillation, based on strain values derived primarily from LV-based software. Given the systematic underestimation in LA strain observed with the AFI-LA package in our study, these cutoffs may not be directly transferable and could lead to misclassification when applied without adjustment. Software specific ranges and recalibration would mitigate these discrepancies, improving the ability to perform comparisons across vendor systems, thereby enhancing the clinical utility of LAS. This would also permit the use of the large repository of clinical data pertaining to LAS that has been previously obtained using the LV strain package.

We propose ‘recalibration’ of AFI-LA measurements to correct for differences observed between GE mid-myocardial, GE endocardial, and TomTec-derived phasic LAS. However, we acknowledge that these adjustments are cohort-specific, and robust recalibration would require formal derivation and validation in independent datasets before clinical application. This may enable more accurate comparisons between software platforms, particularly when serial LAS measurements for a patient are performed on different systems. A precedent for such standardization exists for LV strain measurements, which were aligned through a joint initiative led by the European and American Societies of Echocardiography [8] to eliminate vendor-based differences. Given the increasing clinical importance of LAS, a similar call to action is needed to standardize phasic LA strain measurements across different vendor platforms and importantly, to derive ‘recalibrated’ values using the AFI-LA software platform. This recalibration approach could be integrated into vendor software, where automated correction factors are applied using established conversion formulas.

While our pilot study demonstrates differences in phasic LAS measurements, larger datasets are needed to validate these findings and develop correction factors across diverse populations using different vendor systems. Additionally, comparisons of DICOM versus raw data need to be performed to ensure their accuracy and reliability before widespread clinical implementation. In addition, future collaborative efforts among vendors, international echocardiography societies, and researchers will be crucial for achieving standardization of LAS measurements. Building on the success of LV strain standardization, the development of a universally accepted ‘gold standard’ for LAS measurement—through consensus-driven protocols and large-scale validation studies—could enhance the uptake and clinical utility of LAS. Such efforts would facilitate its broader adoption in routine clinical practice and improve risk stratification in conditions such as atrial fibrillation and heart failure.

## Limitations

There are several limitations of our study. The sample size included was relatively small and, although it included healthy controls as well as paroxysmal AF patients who had enlarged LA volumes, it may not have captured all aspects of variability, especially across different patient populations. However, this is a pilot ‘proof of concept’ study that demonstrates small yet significant differences in phasic LAS measurements. Further studies on larger cohorts would be important to determine whether the relationships between AFI-LA and LAS derived using LV strain packages from various vendors previously used for LA strain measurements can be accurately ‘recalibrated’. Additional analysis, comparing patients with various grades of LA enlargement and diverse disease groups, needs to be performed.

## Conclusion

Our study demonstrated small yet significant differences in LAS measurements between the new AFI-LA package, which is LA specific, and previously used GE mid-myocardial, GE endocardial, and TomTec LV strain packages, utilised to obtain LA strain. The LAS measurements obtained using AFI-LA software are most similar to GE mid-myocardial measurements, with a wider systematic bias when compared with GE endocardial and endocardial feature tracking TomTec LAS. We propose larger studies with derivation and validation groups in diverse patient populations that evaluate recalibration for AFI-LA measurements, thereby permitting comparisons between serial LAS measurements to be undertaken using different algorithms.

## Data Availability

No datasets were generated or analysed during the current study.
